# Supplementary material for: Aberrant gene activation in synovial sarcoma relies on SSX specificity and increased PRC1.1 stability
Source: Nat Struct Mol Biol. 2023 Sep 21;30(11):1640–52. doi: 10.1038/s41594-023-01096-3 (PMC10643139; doi:10.1038/s41594-023-01096-3)
Supplement: Supplementary file 1 — Reporting Summary [file 41594_2023_1096_MOESM1_ESM.pdf]

nature portfolio

Corresponding author(s): Ana Banito

Last updated by author(s): Jul 27, 2023

Reporting Summary

Nature Portfolio wishes to improve the reproducibility of the work that we publish. This form provides structure for consistency and transparency in reporting. For further information on Nature Portfolio policies, see our Editorial Policies and the Editorial Policy Checklist.

Statistics

For all statistical analyses, confirm that the following items are present in the figure legend, table legend, main text, or Methods section.

n/a	Confirmed	
☐	☒	The exact sample size (*n*) for each experimental group/condition, given as a discrete number and unit of measurement
☐	☒	A statement on whether measurements were taken from distinct samples or whether the same sample was measured repeatedly
☐	☒	The statistical test(s) used AND whether they are one- or two-sided<br>*Only common tests should be described solely by name; describe more complex techniques in the Methods section.*
☒	☐	A description of all covariates tested
☒	☐	A description of any assumptions or corrections, such as tests of normality and adjustment for multiple comparisons
☐	☒	A full description of the statistical parameters including central tendency (e.g. means) or other basic estimates (e.g. regression coefficient) AND variation (e.g. standard deviation) or associated estimates of uncertainty (e.g. confidence intervals)
☐	☒	For null hypothesis testing, the test statistic (e.g. *F*, *t*, *r*) with confidence intervals, effect sizes, degrees of freedom and *P* value noted<br>*Give P values as exact values whenever suitable.*
☒	☐	For Bayesian analysis, information on the choice of priors and Markov chain Monte Carlo settings
☒	☐	For hierarchical and complex designs, identification of the appropriate level for tests and full reporting of outcomes
☒	☐	Estimates of effect sizes (e.g. Cohen's *d*, Pearson's *r*), indicating how they were calculated

Our web collection on statistics for biologists contains articles on many of the points above.

Software and code

Policy information about availability of computer code

Data collection	Imaging was done using the Leica Application Suite Software (4.1). Western blots were imaged using Amersham Imager 680. qRT-PCRs using Roche LightCycler480 II. ChIP libraries were sequenced as 75 bp Single-Read on Illumina NextSeq 550 platform High-Output. SS18-SSX and H2AK119ub1 CUT&RUN libraries were sequenced as 75 bp Paired-End reads on Illumina NextSeq 550 platform Mid-Output. HA-eGFP-SS18, HA-eGFP-SS18-SSX1 and HA-eGFP-SSX-C CUT&RUN libraries and Native H2AK119ub1 calibrated ChIP were sequenced as 50bp Paired-End reads on NovaSeq 6K SP. RNA libraries were sequenced on a NovaSeq 6K Paired-End 100 S4
Data analysis	For ChIP-Seq analysis: Raw reads were trimmed for quality and Illumina adapter sequences using Trim Galore! (Galaxy Version 0.6.7+galaxy0), then aligned to the human genome assembly Hg38 using Bowtie2 (Galaxy Version 2.4.2+galaxy0). ChIP signals were normalised to their respective inputs using the pileup function from MACS2 callpeak (Galaxy Version 2.1.1.20160309.6) using corresponding input for background normalization. To visualize ChIP-Seq tracks, normalized bigWig files were generated with Wig/BedGraph-to-bigWig converter (Galaxy Version 1.1.1).<br><br>For the Cut and Run: Paired-end reads were aligned to the T2T or E.coli K12, MG1655 reference genome using Bowtie2 (Galaxy Version 2.4.2 +galaxy0). Genome coverage files were generated using bamCoverage (Galaxy Version 3.5.1.0.0).<br><br>Image analysis was done using the Fiji software (2.9.0).<br>Statistics and graphs were done using Excel (16.75.2) or Prism (9.4.0).<br>R (versions 3.6.0 to 4.2.1; https://www.r-project.org/).<br>Flow Cytometry analysis: FlowJo (v10.9.0)<br>Gene editing efficiency: Tide (v3.3.0)<br>RNA-seq analysis:IDEP.93 http://bioinformatics.sdstate.edu/idep93/

Gene Tiling screen: MAGeCK (0.5.9) and ProTiler: (1.0.2) were run on Python (3.7.0)

I used python 3.7.0

For manuscripts utilizing custom algorithms or software that are central to the research but not yet described in published literature, software must be made available to editors and reviewers. We strongly encourage code deposition in a community repository (e.g. GitHub). See the Nature Portfolio guidelines for submitting code & software for further information.

Data

Policy information about availability of data

All manuscripts must include a data availability statement. This statement should provide the following information, where applicable:

- Accession codes, unique identifiers, or web links for publicly available datasets
- A description of any restrictions on data availability
- For clinical datasets or third party data, please ensure that the statement adheres to our policy

Re-analysed HA-SS18-SSX1 and KDM2B ChIP sequencing data originates from GEO accession number GSE108929. The GEO accession number for all data created in this paper is reported under GSE205955. Genome assembly used for ChIP and Cut&Run was hg38, for RNAseq hg19. Proteomic data is provided as a supplementary table.

Human research participants

Policy information about studies involving human research participants and Sex and Gender in Research.

Reporting on sex and gender	Sex and gender considerations where not relevant for the biological question addressed in this study.
Population characteristics	Patients who underwent surgical excision specimens in the Vancouver General Hospital to which material was archived between 2007 and 2020.
Recruitment	N/A
Ethics oversight	Tissue Microarray construction from anonymized patient primary surgical excision specimens was performed under protocols H18-00524 and H18-02391, approved by the Clinical Research Ethics Board of the University of British Columbia and BC Cancer.

Note that full information on the approval of the study protocol must also be provided in the manuscript.

Field-specific reporting

Please select the one below that is the best fit for your research. If you are not sure, read the appropriate sections before making your selection.

☒ Life sciences ☐ Behavioural & social sciences ☐ Ecological, evolutionary & environmental sciences

For a reference copy of the document with all sections, see nature.com/documents/nr-reporting-summary-flat.pdf

Life sciences study design

All studies must disclose on these points even when the disclosure is negative.

Sample size	The sample size was not predetermined statistically. The sample size, which is listed in the material and method section, was chosen based on expected variance of the experiments and technical limitations.
Data exclusions	No data exclusions was performed.
Replication	All experiments were repeated independently. The number of biological replicates are indicated in the figure legends.
Randomization	Not relevant because the samples were no grouped, as it includes only molecular assays performed in cell lines of known genotype.
Blinding	Blinding was not relevant for this study as there were no prior assumptions about experimental outcomes. All data was collected and processed uniformly regardless of treatment groups

Reporting for specific materials, systems and methods

We require information from authors about some types of materials, experimental systems and methods used in many studies. Here, indicate whether each material, system or method listed is relevant to your study. If you are not sure if a list item applies to your research, read the appropriate section before selecting a response.

Materials & experimental systems

n/a	Involved in the study
☐	☒ Antibodies
☐	☒ Eukaryotic cell lines
☒	☐ Palaeontology and archaeology
☐	☒ Animals and other organisms
☒	☐ Clinical data
☒	☐ Dual use research of concern

Methods

n/a	Involved in the study
☐	☒ ChIP-seq
☒	☐ Flow cytometry
☒	☐ MRI-based neuroimaging

Antibodies

Antibodies used

anti-NanoLuc, R&D Systems, MAB100261
BCoR (C10), Santa Cruz, sc-514576
EED (E4L6E) XP® Rabbit mAb, Cell Signaling, #85322
Ezh2 (D2C9) XP Rabbit mAb, Cell Signaling, #5246
GFP (D5.1) XP® Rabbit mAb, Cell Signaling, #2956
HA-tag, Abcam, #9110
HA-Tag (6E2) Mouse mAB, Cell Signaling, #2367S
HA-Tag (C29F4) Rabbit mAb, Cell Signaling, #3724
Histone H3 (1B1B2), Cell Signaling, #14269
HP1 (E-6), Santa Cruz, sc- 515341
PCGF1 (E-8), Santa Cruz, sc-515371
SS18-SSX (E9X9V) XP, Cell Signaling, #72364
SS18/SSX Antibody, Cell Signaling, #70929
SSX (E5A2C), Cell Signaling, #23855
ß-Actin HRP, Sigma, A3854
SYT (a-10), Santa Cruz, sc-365170
Tri-Methyl-Histone H3 (Lys27) (C36B11), Cell Signaling, #9733
Tri-Methyl-Histone H3 (Lys9) (D4W1U), Cell Signaling, #13969
Ubiquityl-Histone H2A (Lys119) (D27C4) XP® Rabbit mAb, Cell Signaling, #8240
Acetyl-Histone H3 (Lys27) (D5E4) XP, #8173
V5 Tag Monoclonal Antibody (2F11F7), Alexa Fluor 555, Thermofisher, 2F11F7
v5-Probe (E10), Santa Cruz, sc-81594
V5-Tag (E9H8O) mAb, Cell Signaling, #80076
V5-Taq (D3H8Q) Rabbit mAb, Cell Signaling, #13202
Anti-TATA binding protein TBP antibody[mAbcam51841], Abcam, ab300656
SMARCC1/BAF155 (D7F8S) Rabbit mAb, Cell Signaling, #11956
BRM (D9E8B) XP® Rabbit mAb, Cell Signaling, #11966
p300 (D8Z4E), Cell Signaling, #86377S
Brg-1 (G-7), Santa Cruz, sc-17796
ARID1A/BAF250A (D2A8U) Rabbit, Cell Signaling, #12354
BCOR polyclonal antibody, Proteintech, 12107-1-AP

Secondary antibodies
ECL Anti-Mouse IgG
ECL Anti-Rabbit IgG

Validation

All antibodies were validated by the manufacturers. Tag antibodies (HA, V5) have been extensively used in the literature. In addition, the majority of primary antibodies were further validated using target-specific knockouts (PCGF1 E-8, EZH2 D2C9, EEDE4L6E, BRM D9E8B) or over-expression of a tagged proteins (NanoLuc MAB100261, BCOR polyclonal antibody, Proteintech, 12107-1-AP, BCoR (C10), Santa Cruz, sc-514576).
Antibodies against SS18-SSX and SSX (SS18-SSX (E9X9V) XP, Cell Signaling, #72364 SS18/SSX Antibody, Cell Signaling, #70929 SSX (E5A2C), Cell Signaling, #23855) have been validated by Baranov et al (PMID: 32141887).

p300 (D8Z4E), Cell Signaling, #86377S, Brg-1 (G-7), Santa Cruz, sc-17796, ARID1A/BAF250A (D2A8U) Rabbit, Cell Signaling, #12354 have been used previously in many other studies. See for example PMID: 33651988 (EP300), PMID: 35732731 (BRG1) and PMID: 36435834 (ARID1A).

Antibodies used for histone and histone marks have been extensively used in the literature:
Tri-Methyl-Histone H3 (Lys27) (C36B11), Cell Signaling, #9733 (1065 citations)
Tri-Methyl-Histone H3 (Lys9) (D4W1U), Cell Signaling, #13969 (112 citations)
Ubiquityl-Histone H2A (Lys119) (D27C4) XP® Rabbit mAb, Cell Signaling, #8240 (298 citations)
Histone H3 (1B1B2), Cell Signaling, #14269 (137 citations)
HP1 (E-6), Santa Cruz, sc- 515341 (more than 100 citations)

Eukaryotic cell lines

Policy information about cell lines and Sex and Gender in Research

Cell line source(s)

Human synovial sarcoma cell lines: HS-SY-II (RRID:CVCL_8719) and SYO-1 (RRID:CVCL_7146) were obtained fom their original

Cell line source(s)	source laboratories. Human osteosarcoma KHOS-240S (RRID:CVCL_2544) and Human Embryonic Kidney HEK293T (RRID:CVCL_0063) were purchased from the American Type Culture Collection (ATCC). ASC52telo, hTERT immortalized adipose derived Mesenchymal stem cells were purchased from ATCC (SCRC-4000). Drosophila SG-4 cell line used for calibrated ChIP was provided by Angelika Feldmann (German Cancer Research Center).
Authentication	HS-SY-II and SYO-I were authenticated via classical STR profiling with the company Multiplexion and by western blot for SS18-SSX1/2 detection. he remaining cell lines (MSCs, HEK293-T, KHOS-240S and SG-4) were obtained authenticated by the manufacturer (ATCC) and by morphology.
Mycoplasma contamination	Cell lines were monthly tested against mycoplasma contamination and remained negative.
Commonly misidentified lines (See ICLAC register)	No misidentified cell lines were used in this study.

Animals and other research organisms

Policy information about studies involving animals; ARRIVE guidelines recommended for reporting animal research, and Sex and Gender in Research

Laboratory animals	This study used Mus musculus (C57BL/6J) with a conditional SS18-SSX2-IRES-eGFP allele knocked into the Rosa26 locus. Tamoxifen treatment was performed on 8 weeks old mice. Mice were housed under standard conditions (12 h light/dark cycle) and provided food and water ad libitum. Animals were maintained in a controlled environment of between 21-24o C and 40-60% humidity, and experimental protocols were conducted in accordance with approved and ethical treatment standards of the Animal Care Committee at the University of British Columbia.
Wild animals	No wild animals were used.
Reporting on sex	No sex specific data was used.
Field-collected samples	No field-collected samples were used.
Ethics oversight	Animals were maintained and experimental protocols were conducted in accordance with approved and ethical treatment standards of the Animal Care Committee at the University of British Columbia.

Note that full information on the approval of the study protocol must also be provided in the manuscript.

ChIP-seq

Data deposition

☒ Confirm that both raw and final processed data have been deposited in a public database such as GEO.

☒ Confirm that you have deposited or provided access to graph files (e.g. BED files) for the called peaks.

Data access links *May remain private before publication.*	https://www.ncbi.nlm.nih.gov/geo/query/acc.cgi?acc=GSE205955
Files in database submission	GSM6235997 HSSY, eGFP, input<br>GSM6235998 HSSY, eGFP, HA<br>GSM6235999 HSSY_SSX_input<br>GSM6236000 HSSY_SSX_HA<br>GSM6236001 HSSY_SSXDeltaRD_input<br>GSM6236002 HSSY_SSXDeltaRD_HA<br>GSM6236003 HSSY, IgG<br>GSM6236004 HSSY, MacroH2A2<br>GSM6236005 HSSY, Control, H2Aub1<br>GSM6236006 HSSY, Control, HA (SS18-SSX1)<br>GSM6236007 HSSY, PCGF1 knockout, H2Aub1<br>GSM6236008 HSSY, PCGF1 knockout, HA (SS18-SSX1)<br>GSM6236009 SYOI, IgG<br>GSM6236010 SYOI, Control, H2Aub1<br>GSM6236011 SYOI, Control, SS18-SSX2<br>GSM6236012 SYOI, PCGF1 knockout, H2Aub1<br>GSM6236013 SYOI, PCGF1 knockout, SS18-SSX2
Genome browser session (e.g. UCSC)	

Here is the link to the updated bigwigs:
https://dl.dropboxusercontent.com/s/b9ordhxtpcac3gf/HSS_cChIP_EV_H2A.bigwig?dl=0
cCHIP_sgPCGF1_H2A
https://dl.dropboxusercontent.com/s/xlvs7fhu6uwrrk4/HSS_cChIP_sgPCGF1_H2A.bigwig?dl=0
HSS_CR_EV_H2Aub
https://dl.dropboxusercontent.com/s/zdv5a4l7zlkpw01/HSS_CR_EV_H2Aub.bigwig?dl=0
HSS_CR_EV_SS18SSX
https://dl.dropboxusercontent.com/s/htjf4d0n9czyfan/HSS_CR_EV_SS18-SSX.bigwig?dl=0
HSS_CR_sgPCGF1_H2Aub
https://dl.dropboxusercontent.com/s/yi8cjf4lejjxzr5/HSS_CR_sgPCGF1_H2Aub.bigwig?dl=0
HSS_CR_sgPCGF1_SS18SSX
https://dl.dropboxusercontent.com/s/8wfyomyzam0rnev/HSS_CR_sgPCGF1_SS18-SSX.bigwig?dl=0
HSS_CR_SSX_C_DOX
https://dl.dropboxusercontent.com/s/ecklriqyjtyjh68/HSS_CR_SSX-C_Dox.bigwig?dl=0
HSS_CR_SSX_C_NoDox
https://dl.dropboxusercontent.com/s/j4n6197q8p8e0yi/HSS_CR_SSX-C_NoDox.bigwig?dl=0
KHOS_CR_EV
https://dl.dropboxusercontent.com/s/01lv1a8shce6kaz/KHOS_CR_EV.bigwig?dl=0
KHOS_CR_SS18
https://dl.dropboxusercontent.com/s/j4kmdt4su6t9w31/KHOS_CR_SS18.bigwig?dl=0
KHOS_CR_SS18-SSX
https://dl.dropboxusercontent.com/s/mzqs2x99k4grdst/KHOS_CR_SS18-SSX.bigwig?dl=0
KHOS_CR_SSX
https://dl.dropboxusercontent.com/s/rqsxm3e18wuovvd/KHOS_CR_SSX.bigwig?dl=0
SYOI_CR_EV_H2Aub
https://dl.dropboxusercontent.com/s/rbop41e9on1l4im/SYOI_CR_EV_H2Aub.bigwig?dl=0
SYOI_CR_EV_SS18SSX
https://dl.dropboxusercontent.com/s/a022ih0b4jv4fj6/SYOI_CR_EV_SS18-SSX.bigwig?dl=0
SYOI_sgPCGF1_H2Aub
https://dl.dropboxusercontent.com/s/efiruldy66pvvyy/SYOI_CR_sgPCGF1_H2Aub.bigwig?dl=0
SYOI_sgPCG1_SS18SSX
https://dl.dropboxusercontent.com/s/hurzbom8lfdauhn/SYOI_CR_sgPCGF1_SS18-SSX.bigwig?dl=0

Methodology

Replicates

The ChIP sequencing was performed in a unique biological replicate. Cut and Run sequencing of endogenous SS18-SSX and H2AK119ub1 was performed in two independent cell lines HS-SY-II and SYO-I. Native H2AK119ub1 calibrated ChIP was perfomed in biological triplicates. Cut and Run of overexpressed constructs was done in one replicate.

Sequencing depth

ChIP libraries were sequenced as 75 bp Single-Read on Illumina NextSeq 550 platform High-Output.
total reads.
eGFP input: 31465378
eGFP HA: 35710039
SSX-C input: 39821585
SSX-C HA: 35710039
DeltaRD input: 38916894
DetaRD HA: 27970228

CUT&RUN libraries were sequenced as 75 bp Paired-End reads on Illumina NextSeq 550 platform Mid-Output.
total reads:
HSS IgG: 13526884
HSS EV H2Aub: 19510962
HSS EV SS18-SSX: 11610526
HSS sgPCGF1 H2Aub: 15938066
HSS sgPCGF1 SS18-SSX: 13320300
SYOI IgG: 23814054
SYOI EV H2Aub: 23120676
SYOI EV SS18-SSX: 20301480
SYOI sgPCGF1 H2Aub: 25397092
SYOI sgPCGF1 SS18-SSX: 15248120

CUT&RUN for overexpression were sequenced as 50bp Paired-End reads on NovaSeq 6K SP.
total reads:
KHOS IgG 27488012
KHOS EV 55388126
KHOS SS18 45457932
KHOS SS18-SSX 41549600
KHOS SSX-C 46110250

Native H2AK119ub1 calibrated ChIP were sequenced as 50bp Paired-End reads on NovaSeq 6K SP.
total reads:
HSS EV R1 23109716
HSS EV R2 29247779:
HSS EV R3 29882136:
HSS sgPCF1 R1 29064591

HSS sgPCF1 R2 39902391
HSS sgPCF1 R3 49357058

Antibodies

The ChIP sequencing has been done using the HA-tag antibody #9110 from Abcam.
For the Cut and Run we used HA-Tag (C29F4) Rabbit mAb #3724 Cell Signaling,
SS18-SSX (E9X9V) XP #72364 Cell Signaling, Ubiquityl-Histone H2A (Lys119) (D27C4) XP® Rabbit mAb #8240 Cell Signaling.
Native H2AK119ub1 calibrated ChIP was done using Ubiquityl-Histone H2A (Lys119) (D27C4) XP® Rabbit mAb #8240 Cell Signaling.

Peak calling parameters

For ChIP sequencing HA-SS18-SSX1 peaks (n=26805) were generated with the MACS2 function (with "--no model", "--qvalue 0.05", "--broad" options) and normalized to input.
For H2AK119ub1 (n=11099) and SS18-SSX2 (n= 27686) peak calling, the MACS2 callpeak function was used on the aligned BAM files and IgG as control (with "--nomodel", "--qvalue 0.01", "--broad" options, "--keep-dup all"). For HA peak calling in KHOS-240S, HA-SS18, HA-SS18-SSX1 and HA-eGFP-SSX1 were combined in MACS2 to compute all the HA peaks (n=58843).

Data quality

We assessed the quality of the sequencing using FastQC and Plotfingerprint. For Cut and Run, the percentage of reads mapped to E.Coli was also an indication of the success of assay. As an indication, histone H2Aub pull down has a percentage of E.Coli reads between 0.5 and 1. For SS18-SSX the percentage is between 1.5 and 3.5 % and IgG around 15%. Therefore a Cut and Run sequencing that shows abnormally high E.Coli content is a sign that the pull down did not work.

Software

We used Galaxy program for the analysis of the ChIP sequencing and the Cut and Run.
For ChIP sequencing: Raw reads were trimmed for quality and Illumina adapter sequences using Trim Galore! (Galaxy Version 0.6.7 +galaxy0), then aligned to the human genome assembly Hg38 using Bowtie2 (Galaxy Version 2.4.2+galaxy0). ChIP signals were normalised to their respective inputs using the pileup function from MACS2 callpeak (Galaxy Version 2.1.1.20160309.6) using corresponding input for background normalization. To visualize ChIP-Seq tracks, normalized bigWig files were generated with Wig/BedGraph-to-bigWig converter (Galaxy Version 1.1.1).
For the Cut and Run: Paired-end reads were aligned to the T2T or E.coli K12, MG1655 reference genome using Bowtie2 (Galaxy Version 2.4.2+galaxy0). Genome coverage files were generated using bamCoverage (Galaxy Version 3.5.1.0.0).
