# Supplementary material for: Aberrant gene activation in synovial sarcoma relies on SSX specificity and increased PRC1.1 stability
Source: Nat Struct Mol Biol. 2023 Sep 21;30(11):1640–52. doi: 10.1038/s41594-023-01096-3 (PMC10643139; doi:10.1038/s41594-023-01096-3)

Figure 1j

SS18-SSX + Precision Plus Protein Dual Color Standards

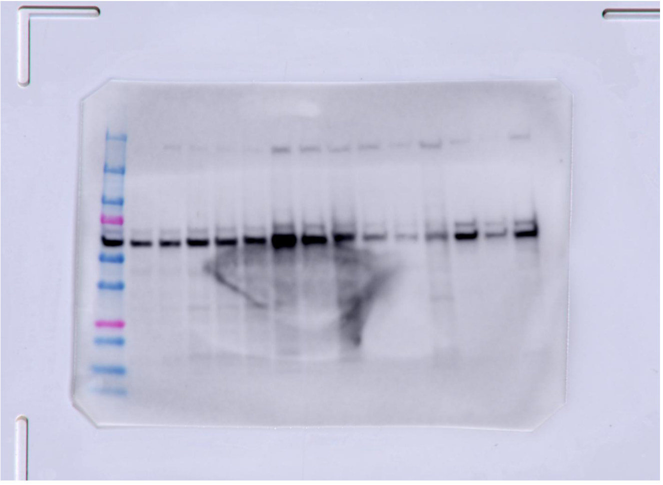

SS18-SSX

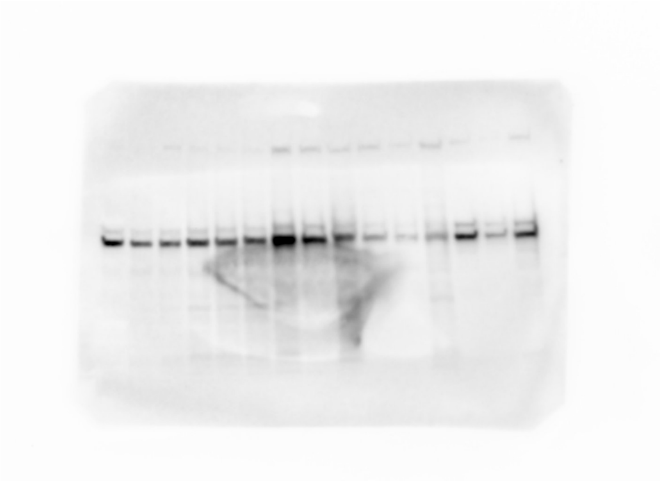

β-Actin + Precision Plus Protein Dual Color Standards

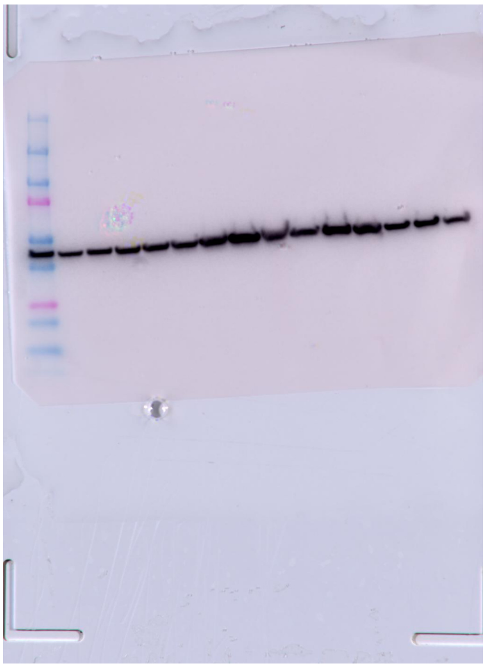

β-Actin

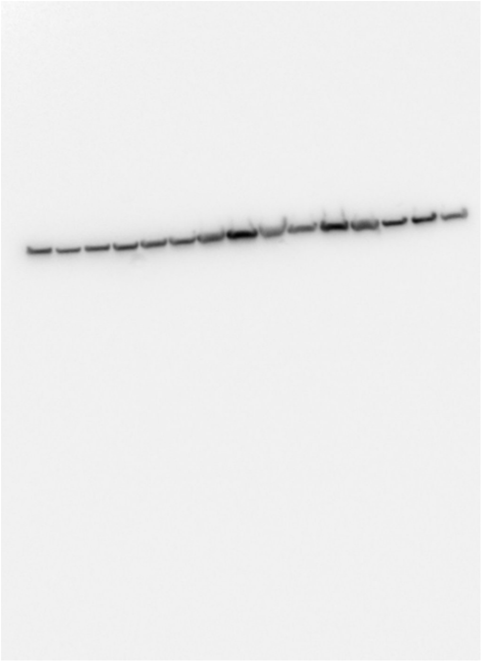

Supplement: Supplementary file 5 — Unprocessed western blots. [file 41594_2023_1096_MOESM5_ESM.pdf]
