# Supplementary figures and images for: Aberrant gene activation in synovial sarcoma relies on SSX specificity and increased PRC1.1 stability
Source: Nat Struct Mol Biol. 2023 Sep 21;30(11):1640–52. doi: 10.1038/s41594-023-01096-3 (PMC10643139; doi:10.1038/s41594-023-01096-3)

Figure 3f

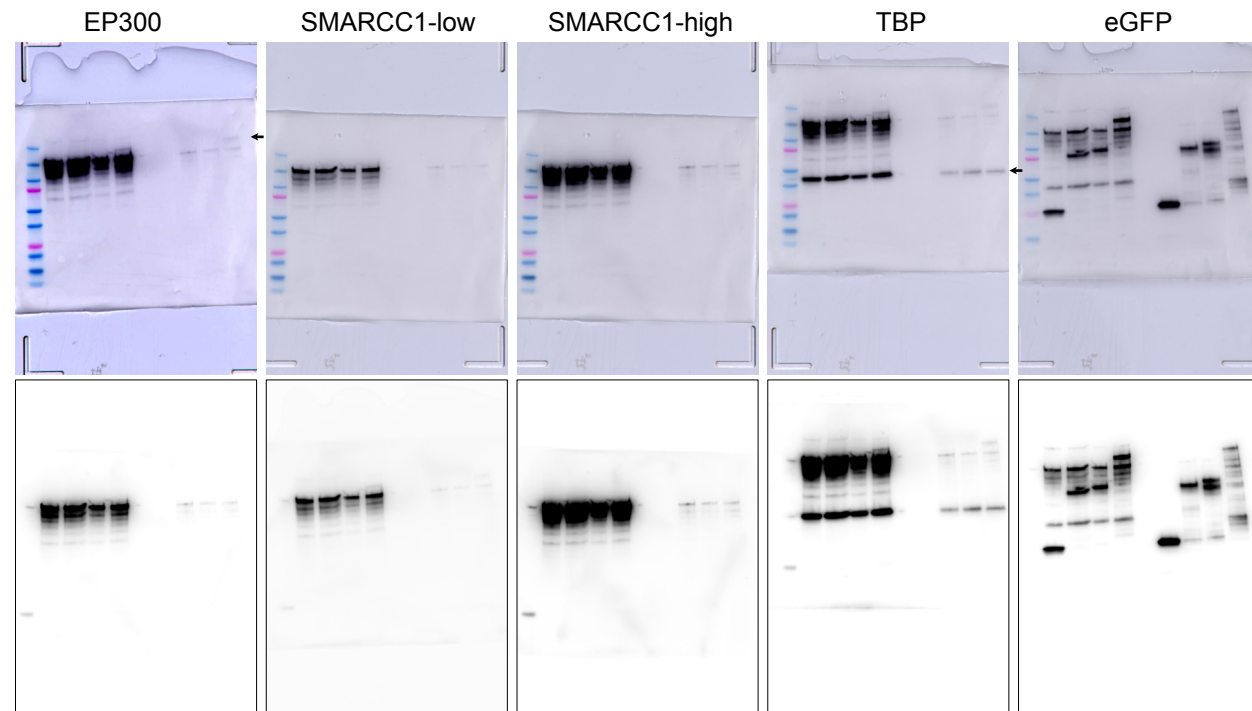

Figure 3g

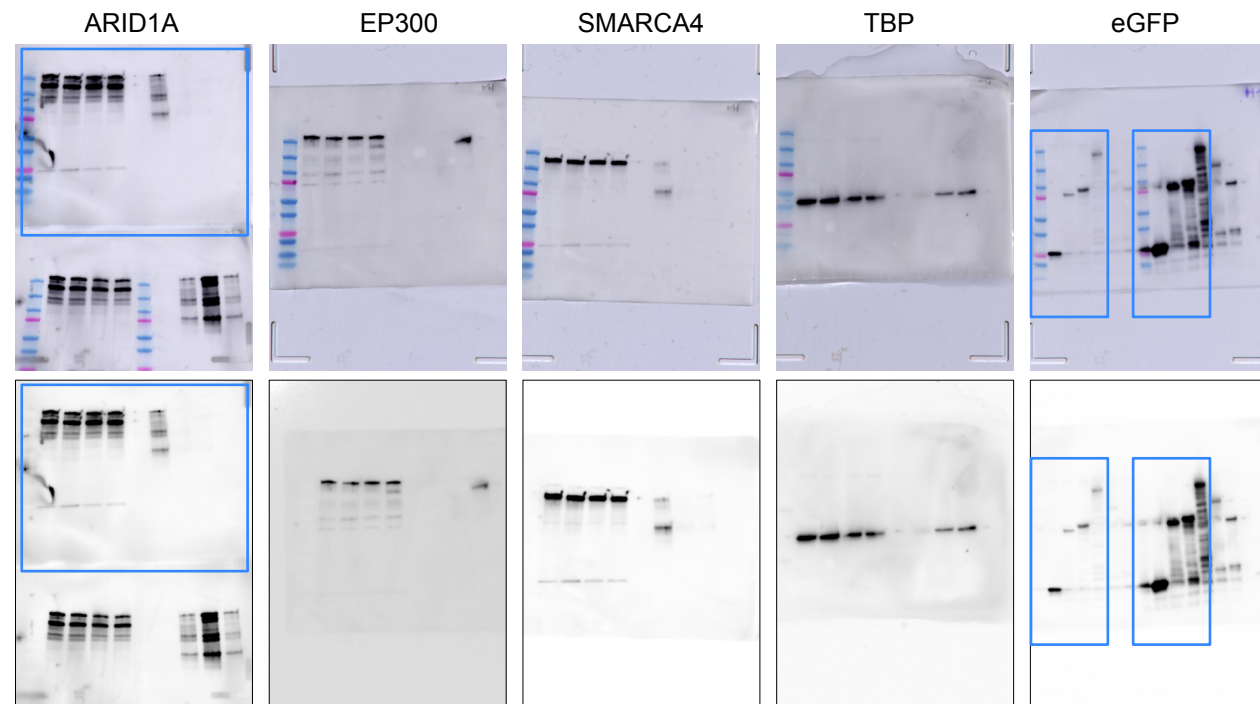

Figure 3h

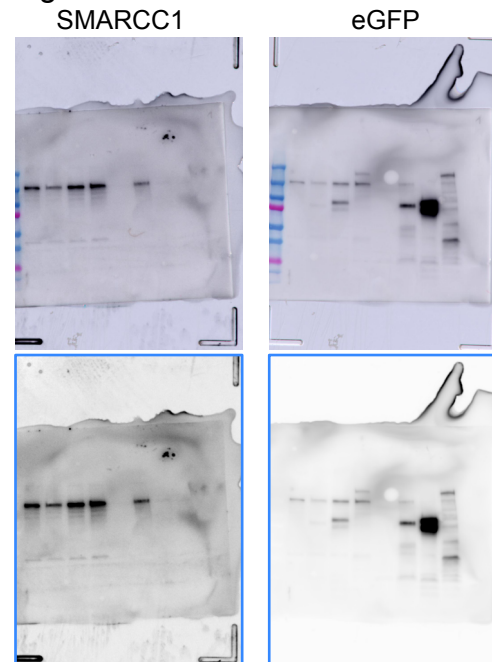

Figure 3i

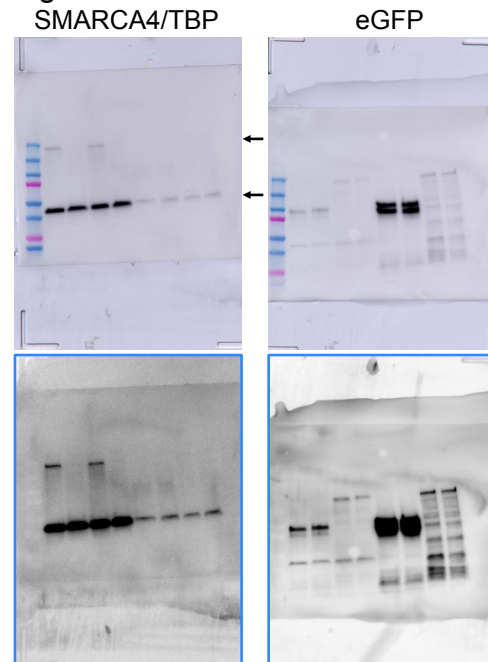

Supplement: Supplementary file 8 — Unprocessed western blots. [file 41594_2023_1096_MOESM8_ESM.pdf]

Figure 4b

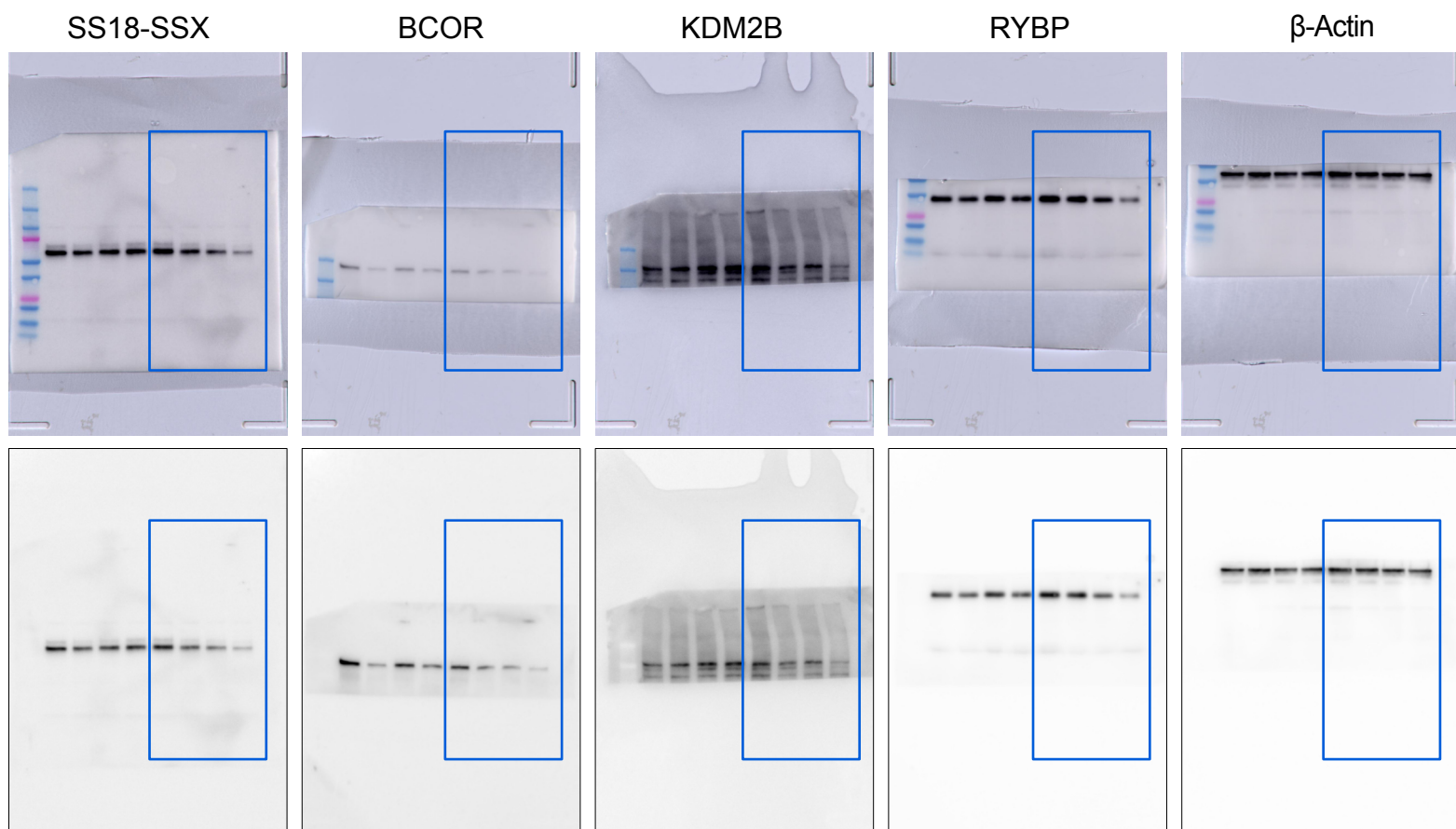

Figure 4g

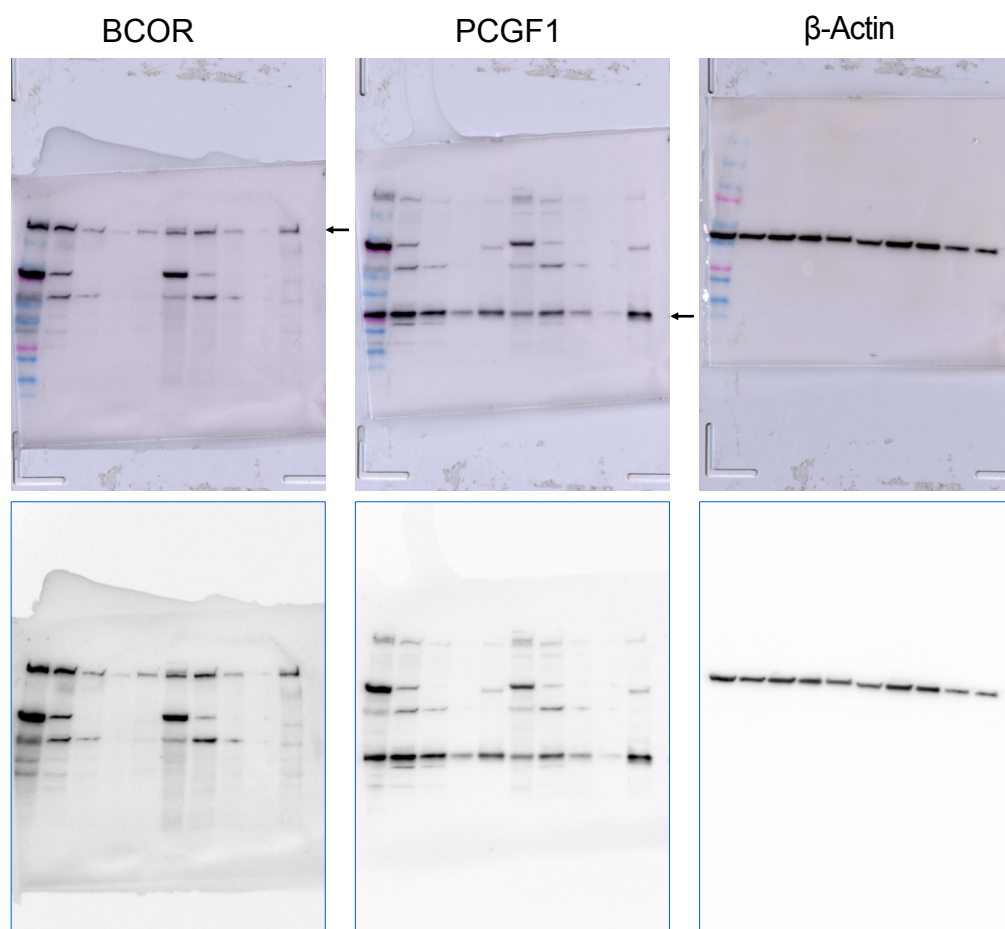

Supplement: Supplementary file 10 — Unprocessed western blots. [file 41594_2023_1096_MOESM10_ESM.pdf]

Extended Figure 1c

BCOR, EZH2, EED, PCGF1

BCOR →  
EZH2 →  
EED →  
PCGF1 →

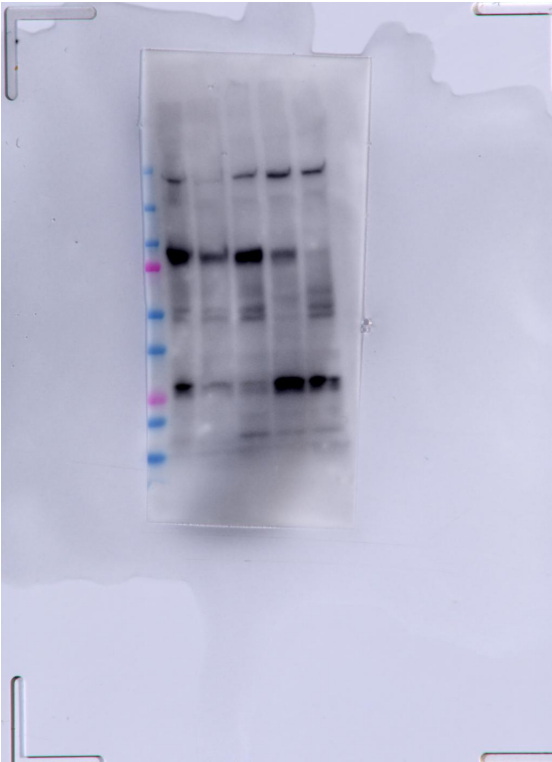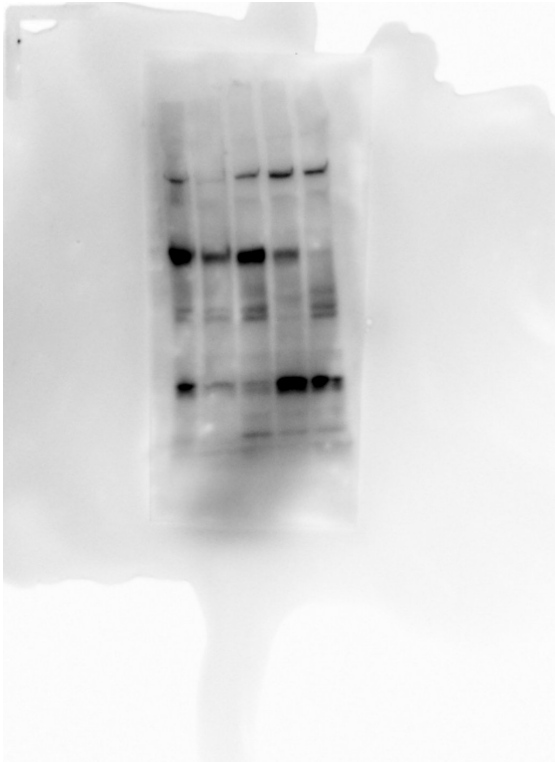

β-Actin

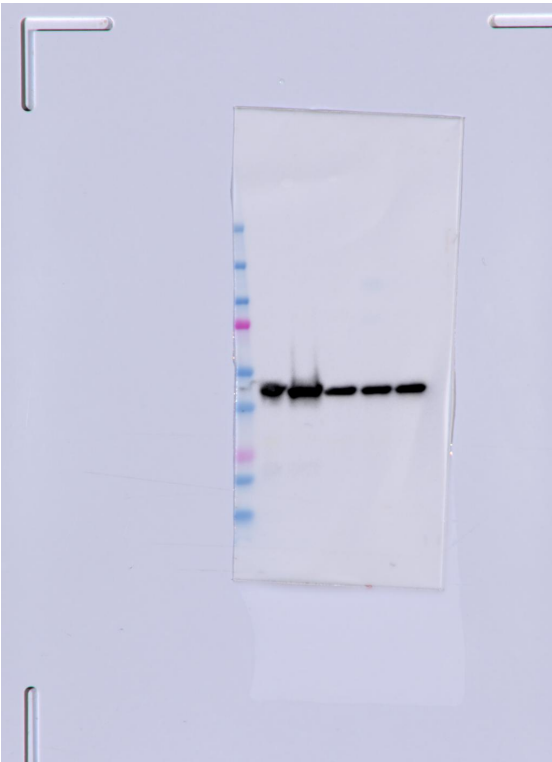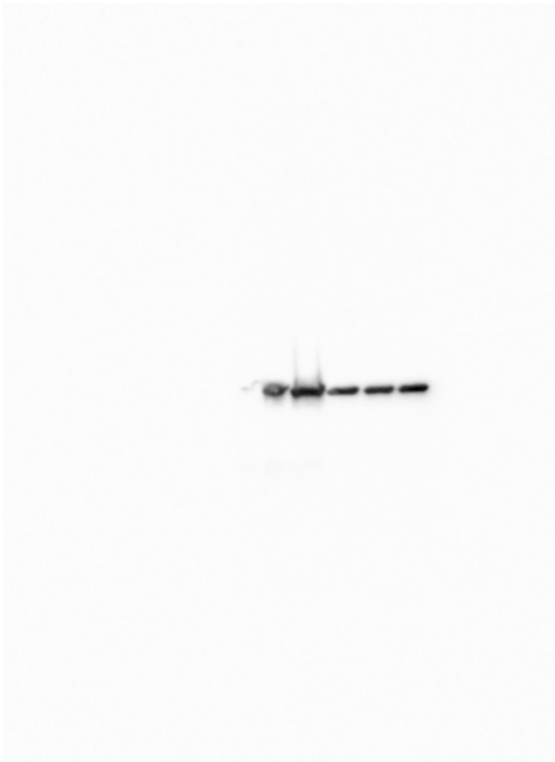

Supplement: Supplementary file 13 — Unprocessed western blots. [file 41594_2023_1096_MOESM13_ESM.pdf]
