# Supplementary material for: Aberrant gene activation in synovial sarcoma relies on SSX specificity and increased PRC1.1 stability
Source: Nat Struct Mol Biol. 2023 Sep 21;30(11):1640–52. doi: 10.1038/s41594-023-01096-3 (PMC10643139; doi:10.1038/s41594-023-01096-3)

Extended Figure 2c

Blot Left: eGFP, SS18 and SS18-SSX

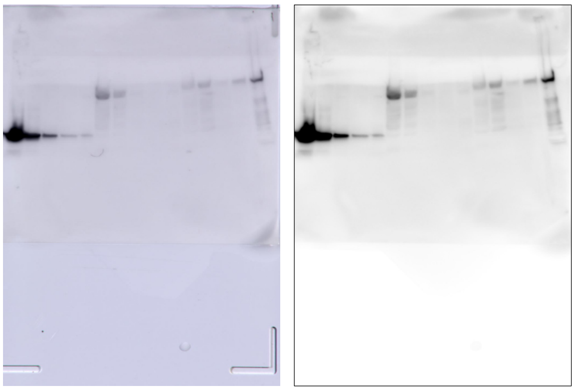

Blot Right: SSX-C, SSXRD, SSXC-DeltaRD

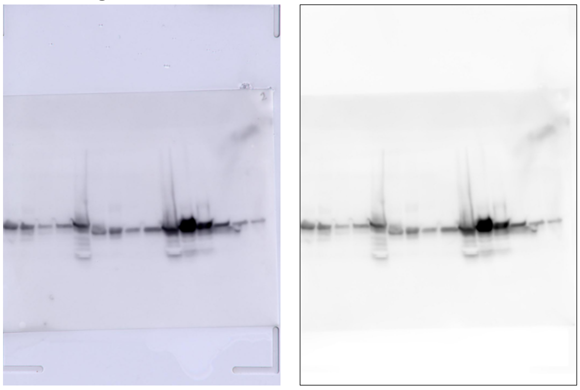

Extended Figure 2f

NLuc

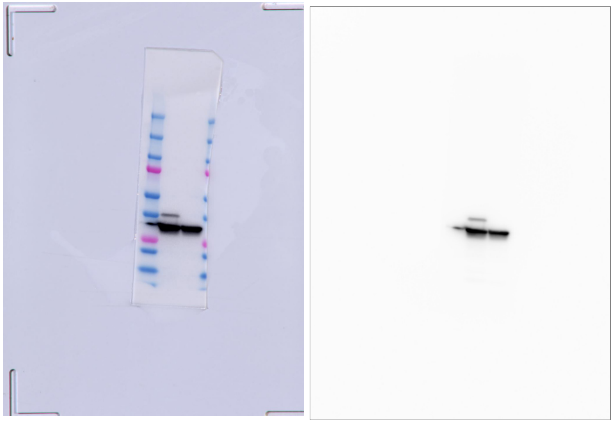

H2AK119ub1

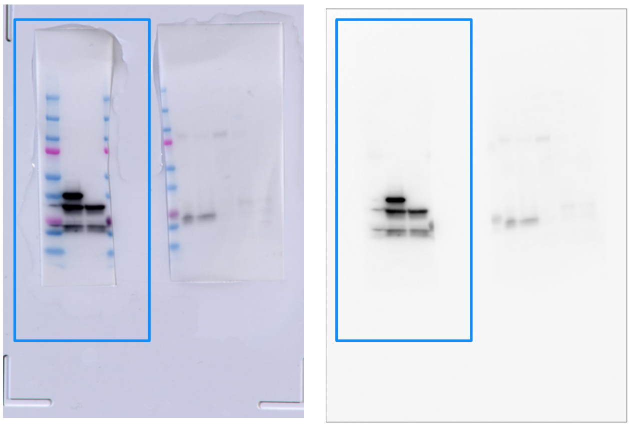

Extended Figure 2h

SS18-SSX

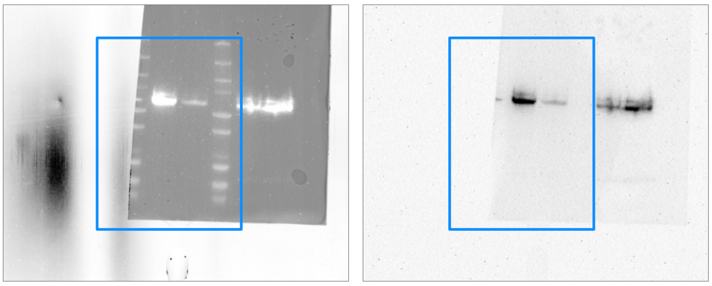

β-Actin

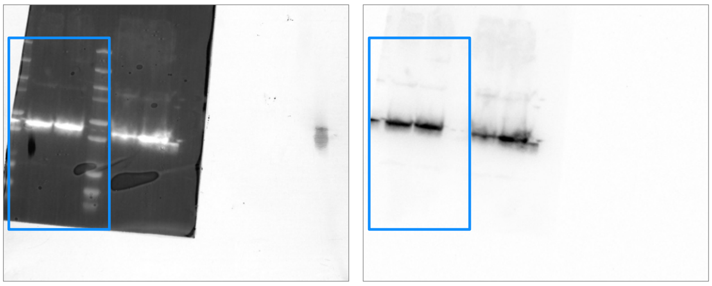

Extended Figure 2j

SMARCA4

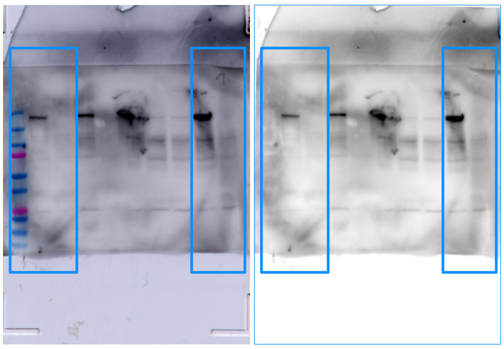

SMARCC1

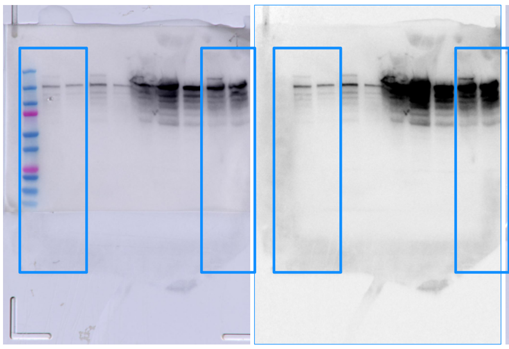

β-Actin

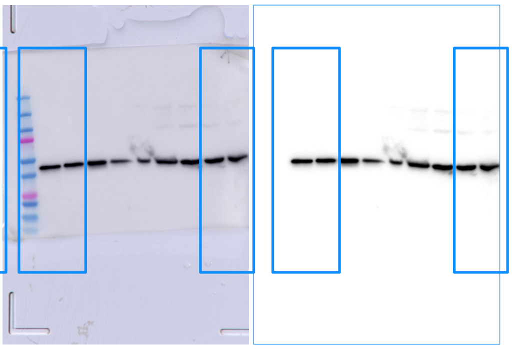

Supplement: Supplementary file 15 — Unprocessed western blots. [file 41594_2023_1096_MOESM15_ESM.pdf]
