# Supplementary material for: Aberrant gene activation in synovial sarcoma relies on SSX specificity and increased PRC1.1 stability
Source: Nat Struct Mol Biol. 2023 Sep 21;30(11):1640–52. doi: 10.1038/s41594-023-01096-3 (PMC10643139; doi:10.1038/s41594-023-01096-3)

Extended Figure 4c

SS18-SSX1 PCGF1 β-Actin

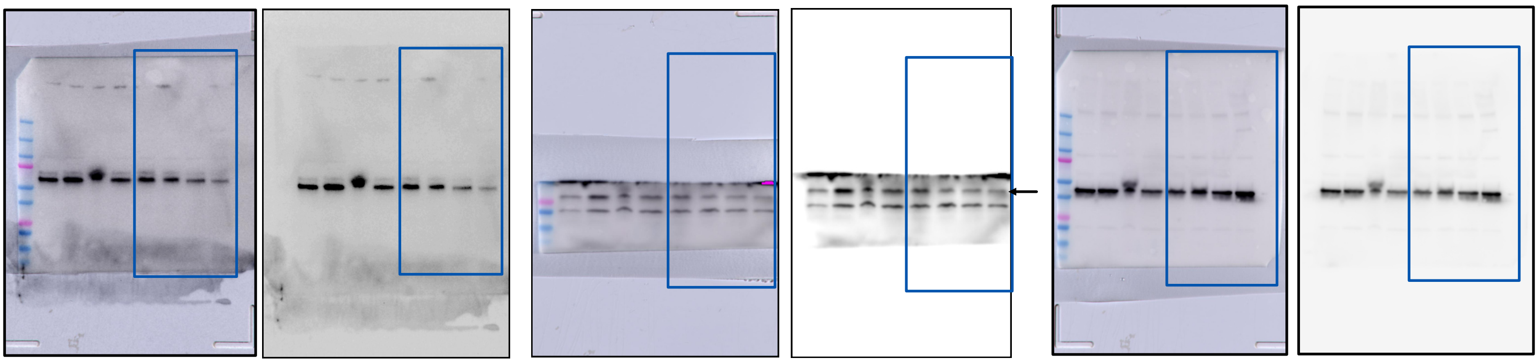

Extended Figure 4f

BCOR/PCGF1 β-Actin

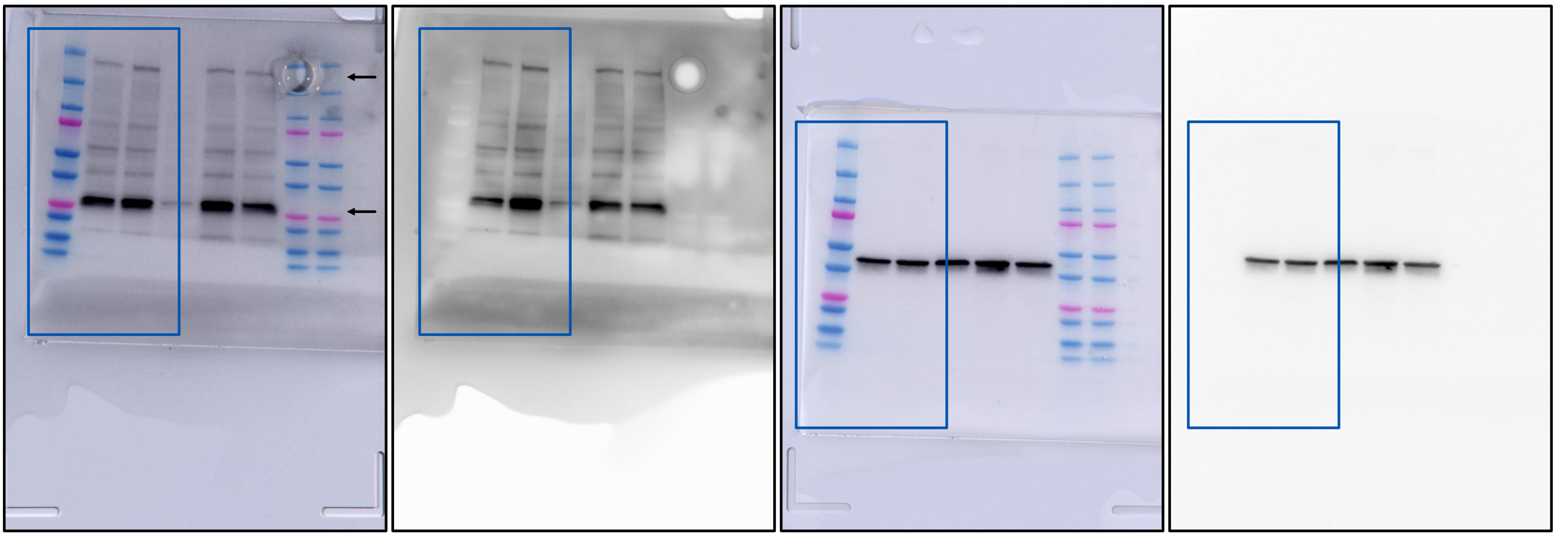

Extended Figure 4g

BCOR PCGF1 β-Actin

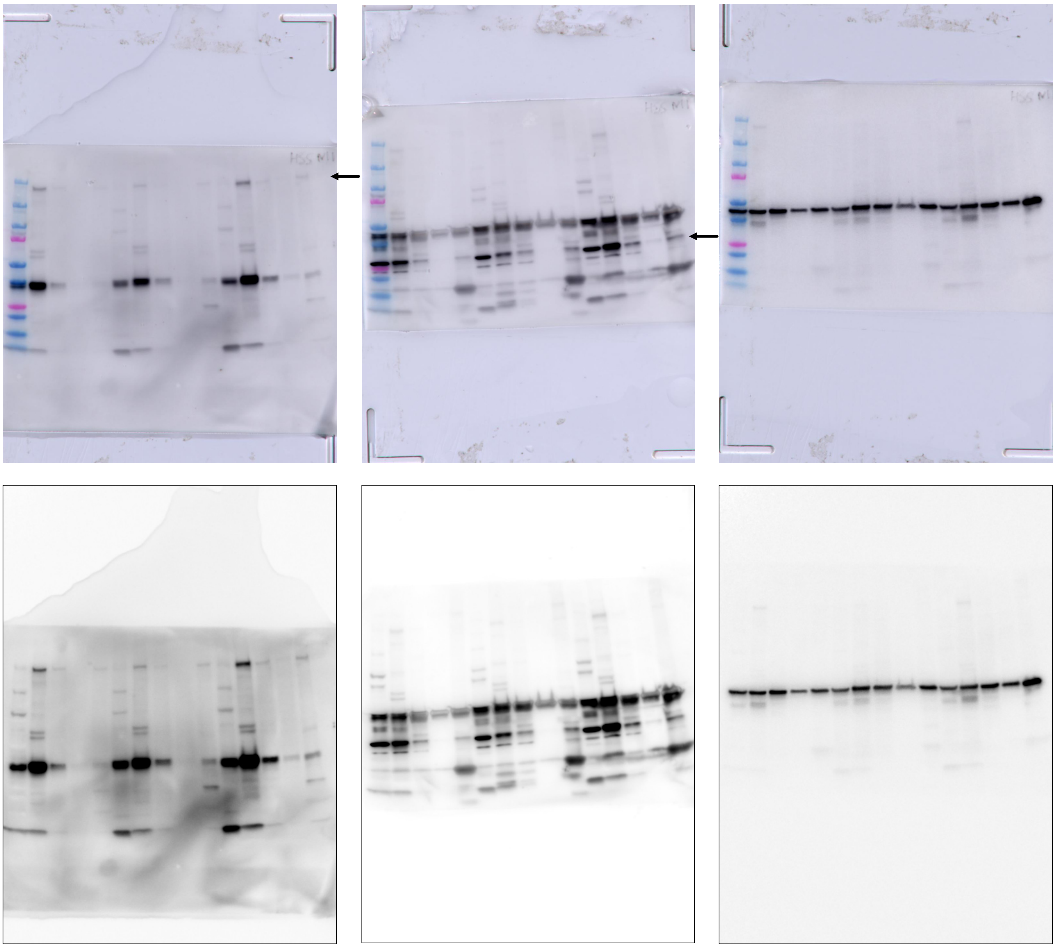

Supplement: Supplementary file 18 — Unprocessed western blots. [file 41594_2023_1096_MOESM18_ESM.pdf]
